# Enhanced Bioavailability of Dihydrotanshinone I–Bovine Serum Albumin Nanoparticles for Stroke Therapy

**DOI:** 10.3389/fphar.2021.721988

**Published:** 2021-08-31

**Authors:** Yanru Ren, Yicheng Feng, Kunyao Xu, Saisai Yue, Tiantian Yang, Kaili Nie, Man Xu, Haijun Xu, Xin Xiong, Fabian Körte, Mike Barbeck, Peisen Zhang, Luo Liu

**Affiliations:** ^1^ Beijing Advanced Innovation Center for Soft Matter Science and Engineering, College of Life Science and Technology, Beijing University of Chemical Technology, Beijing, China; ^2^ NMI Natural and Medical Sciences Institute at the University of Tübingen, Reutlingen, Germany; ^3^ Institute of Material Science and Technology, Technical University of Berlin, Berlin, Germany

**Keywords:** dihydrotanshinone I, stroke therapy, nanoparticles, bioavailability, encapsulation

## Abstract

Dihydrotanshinone I (DHT) is a natural component in *Salvia miltiorrhiza* and has been widely researched for its multiple bioactivities. However, poor solubility and biocompatibility of DHT limit its desirable application for clinical purposes. Herein, DHT was encapsulated with bovine serum albumin (BSA) to enhance bioavailability. Compared to free DHT, DHT–BSA NPs (nanoparticles) showed an improved solubility in normal saline and increased protection against hydrogen peroxide–induced oxidative damage in PC12 cells. In addition, DHT–BSA NPs administered by intravenous injection displayed a significant efficacy in the middle cerebral artery occlusion/reperfusion models, without any impact on the cerebral blood flow. In summary, DHT–BSA NPs show an enhanced bioavailability compared with free DHT and a successful penetration into the central nervous system for stroke therapy, demonstrating their application potential in cardio–cerebrovascular diseases.

## Introduction

According to previous studies, dihydrotanshinone I (DHT), as a kind of active ingredient in the roots of *Salvia miltiorrhiza* Bunge, can play a variety of functions in the treatment of ischemic stroke (Park et al., 2012; Zhang et al., 2013a; Lin et al., 2015; Zhu et al., 2021), including dilating blood vessels (Zhou et al., 2011), improving blood microcirculation (Yue et al., 2021), antiplatelet aggregation (Wang et al., 2020a), reducing blood viscosity, scavenging reactive oxygen species (Ge et al., 2017), anti-inflammatory activities (Wei et al., 2016), and protecting vascular endothelial cells (Wang et al., 2020b). However, the aqueous solubility and biocompatibility of this molecule are rather low, which largely hampers its biological applications (D’Agostino et al., 2015; Ye et al., 2012; Xu et al., 2020). Besides, owing to its low molecular weight, this small-molecule drug has a short blood half-life and it is difficult to guarantee the local concentration of the drug after intravenous administration (Gao et al., 2018; Carriles et al., 2021). Even worse, the presence of the blood–brain barrier (BBB) further impairs the therapeutic effect of this agent after stroke (Sadeghi et al., 2014).

To enhance the solubility and bioavailability of such kind of poorly water-soluble drugs, some effective efforts have been made (Li et al., 2020). For example, in some commercial pharmaceutical preparations, the surfactant and cosolvents such as Tween 80 and ethanol are employed to solubilize the hydrophobic drugs, which successfully obtain the pharmaceutical preparations that can fulfill the requirements for intravenous injection (Kawakami et al., 2006; He et al., 2010). In many laboratory research studies, the inorganic mesoporous nanomaterials were also used to load the hydrophobic drugs, which can not only provide a solubility enhancement of the drugs but also control the drug release rate (Zhang et al., 2019; Lin et al., 2021). However, the pharmacokinetics and elimination pathways of these exogenous chemicals are difficult to control, thereby leading to the drug additive–related toxicity, which may further cause adverse side effects of the bodies such as irritation or allergy (Souney et al., 2010; Pandey and Kohli, 2018; Dai et al., 2021).

Serum albumin is one of the major soluble proteins in the blood plasma (Dong et al., 2011; Ghorbani et al., 2018; Wang et al., 2020c), which is capable of making complexes with various nutrients with biological activities, delivering them to tissues, and consequently improving their bioavailability (Liang et al., 2019; Tian et al., 2020). Notably, bovine serum albumin (BSA) and human serum albumin (HSA) have as high as approx. 76% sequence homology, and the 3D structure of BSA is reported to be similar to that of HSA (Hou et al., 2017; Kim et al., 2017; Wang et al., 2017). Here, BSA was introduced to enhance the bioavailability of DHT. Due to the residues of hydrophobic amino acids such as Ala and Ile in BSA, the DHT is expected to combine with BSA through hydrophobic interaction, thereby largely improving the aqueous solubility and biocompatibility (Nosrati et al., 2018; Amani et al., 2019; Yang et al., 2019). In addition, benefitting from the nanostructure, BSA is significantly promising in changing the pharmacokinetics of low-molecular-weight drugs, thus prolonging the blood half-life of the DHT, which endows it with a greater probability of crossing the damaged BBB and accumulating in the area of the damaged region after brain stroke (Dubey et al., 2017; Wu et al., 2020; Uma Maheswari et al., 2021).

**SCHEME 1:**
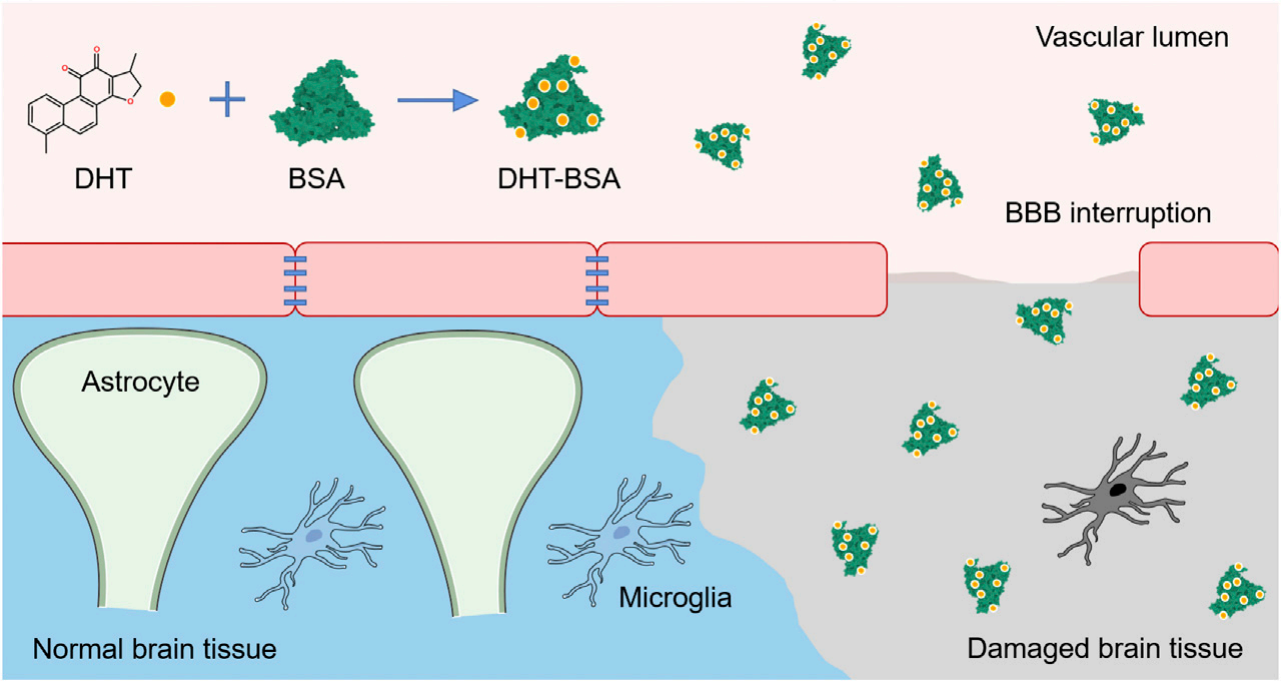
Illustration showing that BBB is permeable and nanoparticles enter the ischemic penumbra after acute ischemic stroke.

In this research, we report for the first time the incorporation of DHT in the BSA nanoparticles, as shown in Scheme 1. The present work focuses on the biosafety, antioxidant ability, and brain delivery capability of DHT-BSA-NPs through the study of cell viability, peroxidase levels *in vitro*, and stroke therapeutic efficacy *in vivo*.

## Materials and Methods

### Materials

Dihydrotanshinone I (CAS: 87205-99-0) was bought from Aladdin (Shanghai, China). Bovine serum albumin (A8010), 2% TTC staining solution (G3005), and dimethyl sulfoxide (V900090) were bought from Solarbio (Shenzhen, China). Superoxide dismutase (SOD), catalase (CAT), glutathione (GSH), and malondialdehyde (MDA) kits were purchased from Nanjing Jiancheng Bioengineering Institute (Jiangsu, China).

### Synthesis of Dihydrotanshinone I–Bovine Serum Albumin Nanoparticles

The DHT–BSA NPs were prepared by the coprecipitation method (Shimanovich et al., 2011; Tabibiazar et al., 2019). Specifically, BSA was dissolved in an aqueous solution and DHT was dissolved in DMSO (Arriagada et al., 2019). Then, the DHT solution was slowly poured into the BSA solution at 1:100 mass ratio of DHT to BSA. The reaction was maintained at room temperature with 4 h of stirring. The resulting nanoparticle solution was purified with 30K MWCO centrifugal devices to remove the free DHT molecules, then transferred into normal saline, and finally stored at 4°C for further use.

### The Characterization of Aqueous Solubility, Absorption Spectrum, Dynamic Light Scattering, and Zeta Potential of Dihydrotanshinone I–Bovine Serum Albumin Nanoparticles

To display the increased water solubility, the DHT–BSA NPs in the aqueous solution were added in a cuvette right after the preparation and photographs were captured. Then, the UV-Vis absorption spectra were recorded at room temperature using a UV-Vis spectrophotometer (ThermoFisher, NanoDrop OneC). Dynamic light scattering (DLS) measurements were conducted on the Zetasizer Nano instrument (Malvern Instruments) equipped with a 10-mW helium–neon laser (λ = 632.8 nm) and thermoelectric temperature controller. The measurements were taken at 25°C with a 90° scattering angle. The sizes and the standard derivations of BSA and DHT–BSA NPs were obtained by averaging the values of at least three measurements. BSA and DHT–BSA were dissolved in ultrapure water and analyzed in an electrophoresis cell at a fixed potential of ±150 mV.

### Cell Culture

PC12 cells (1 × 10^5^) were cultured (100 µl/well in 96-well plates) in Dulbecco’s modified Eagle's medium supplemented with 10% fetal calf serum, 1% penicillin and streptomycin at 37°C, and 5% CO_2_ and 95% air for 24 h (Chang et al., 2021).

### Cell Viability Assay

Cell viability was measured using a 2-(2-methoxy-4-nitrophenyl)-3-(4-nitrophenyl)-5-(2,4-disulfonic acid benzene)-2H-tetrazole monosodium salt (CCK-8) assay. PC12 cells were cultured in the growth medium for 24 h, followed by treatment with various concentrations (0, 62.5, 125, 250, 500, and 1,000 μM) of H_2_O_2_ for 12 h to construct the PC12 cell injury model. 10 μl of CCK-8 solution was added to each well, followed by incubation for 1 h. Cell viability was measured at 450 nm using a Synergy H1 microplate reader. Each treatment was performed in triplicate.

Cells were used for experiments during the exponential growth phase. PC12 cells were preconditioned with different concentrations of DHT–BSA NPs (2.5, 5 and 10 μg/ml) for 12 h, whereas the control cells received 0.9% saline (Beyotime Institute of Biotechnology, Nantong, China) instead (SoukhakLari et al., 2018). Subsequently, PC12 cells were exposed to H_2_O_2_ (500 μM, final concentration) for 12 h.10 μl of CCK-8 solution was added to each well, followed by incubation for 1 h. Cell viability was measured at 450 nm using a Synergy H1 microplate reader. Each treatment was performed in triplicate.

### Antioxidant Level Test

Cells were harvested by centrifugation at 1,000 r/min at 4°C for 5 min, washed with cold phosphate–buffered saline (Gibco Life Technologies; Thermo Fisher Scientific, Inc., Waltham, MA, United States) twice, and homogenized in a lysis buffer containing 20 mM Tris (pH 7.5), 150 mM NaCl, 1% Triton X-100, and 1 mM PMSF. The supernatant was then collected. The levels of SOD, CAT, GSH, and MDA were measured according to the manufacturer's instructions of the respective kits (Nanjing Jiancheng Bioengineering Institute).

### Animals

Male Sprague–Dawley rats (purchased from Beijing Vital River Laboratories, Beijing, China) weighing 250–280 g were used in this study. All rats’ care and experimental procedures were reported in accordance with the Laboratory Animal Ethics Committee of the Institute of Medicinal Plant Development, Peking Union Medical College, and complied with NIH Guidelines for the Care and Use of Laboratory Animals (approval number: SYXK 2017–0020). All rats were maintained in ventilated cages at a temperature of 20–25°C and a relative humidity of 30–50% under a 12 h light–dark cycle and were given free access to food and water.

### Middle Cerebral Artery Occlusion/Reperfusion Surgery

The Sprague–Dawley rats were anesthetized with ketamine (80 mg kg^−1^) and xylazine (10 mg kg^−1^) intraperitoneally by using the middle cerebral artery occlusion/reperfusion (MCAO/R) procedure. Cerebral I/R was induced by MCAO/R as previously described (Voicescu et al., 2017). After MCAO/R surgery, the wound was disinfected with iodine, and then, the wound was sutured with sterile surgical suture to reduce the bleeding. We also injected tramadol (2.5 mg kg^−1^) by tail vein intravenous injection to relieve the pain caused by the operation. The sham operated rats were manipulated using the same surgical procedure, but the middle cerebral artery was not occluded. The body temperature was maintained at 37 ± 0.5°C until rats woke up using a heating pad (Sunbeam, United States). The researcher who conducted all the subsequent analyses was blinded to the treatment that the rats had received.

### *In Vivo* Treatment of Stroke

30 rats were divided into three groups (*n* = 10 per group) according to the random number table. One group of them was set as the sham operation group. And, the other two groups were administered with DHT–BSA NPs or free DHT, respectively. These two drugs were injected intravenously into the rats through the tail vein with the same dose of DHT (1.6 mg kg^−1^) containing an equal volume of physiological saline at the first, third, and fifth days after MCAO/R surgery, respectively. The treatment course lasted for 7 days.

### Neurological Score

Neurological behavior was investigated at 24 h after ischemia/reperfusion (I/R) by two blinded investigators using a 5-point scale as previously published (Voicescu et al., 2017). The neurological function was scored according to a series of scales from 0 to 4 (Lin et al., 2015). The highest score represents the most severe neurological deficits.

### Detection of the Cerebral Blood Flow

The brain cerebral blood flow (CBF) was evaluated using a noninvasive laser-Doppler spectrophotometry system (RWD Life Science Co., Ltd., Guangzhou, China). In brief, the rats were anaesthetized in a calibrated vaporizer with 2% isoflurane/oxygen and an anaesthetic mask delivering 1.5% isoflurane/oxygen. The rats were placed in a supine position on a heating pad with a circulating warm water pump to maintain the body temperature at 37°C. The top of the cranium was shaved, and the CBF was detected using a noninvasive laser-Doppler spectrophotometry system.

### Cerebral Vascular Optical Correlation Tomography Imaging Detection

After treatment, two rats were randomly selected from the DHT–BSA NPs group and the DHT group, respectively, for optical correlation tomography (OCT) detection. After intraperitoneal injection of 1% sodium pentobarbital solution (40 mg kg^−1^ body weight) to anaesthetize the animal, the head of the rat was fixed on a stereotaxic device to expose the skull and a hole was punched on it for OCT imaging detection.

### TTC Staining

TTC staining was conducted 7 days after I/R based on previously described methods (*n* = 5 for each group) (Voicescu et al., 2017). The cerebral infarct area was quantified with an image-analysis system (Image-Pro Plus 5.0). The infarct volume can be obtained by multiplying the total infarct area by the thickness of the brain sections. Calculating the corrected infarct volume contributes to compensating for the error caused by brain edema (Voicescu et al., 2017).

## Results

### The Solubility, Zeta Potential, and Hydrodynamic Size Characterization of Nanoparticles

**FIGURE 1:**
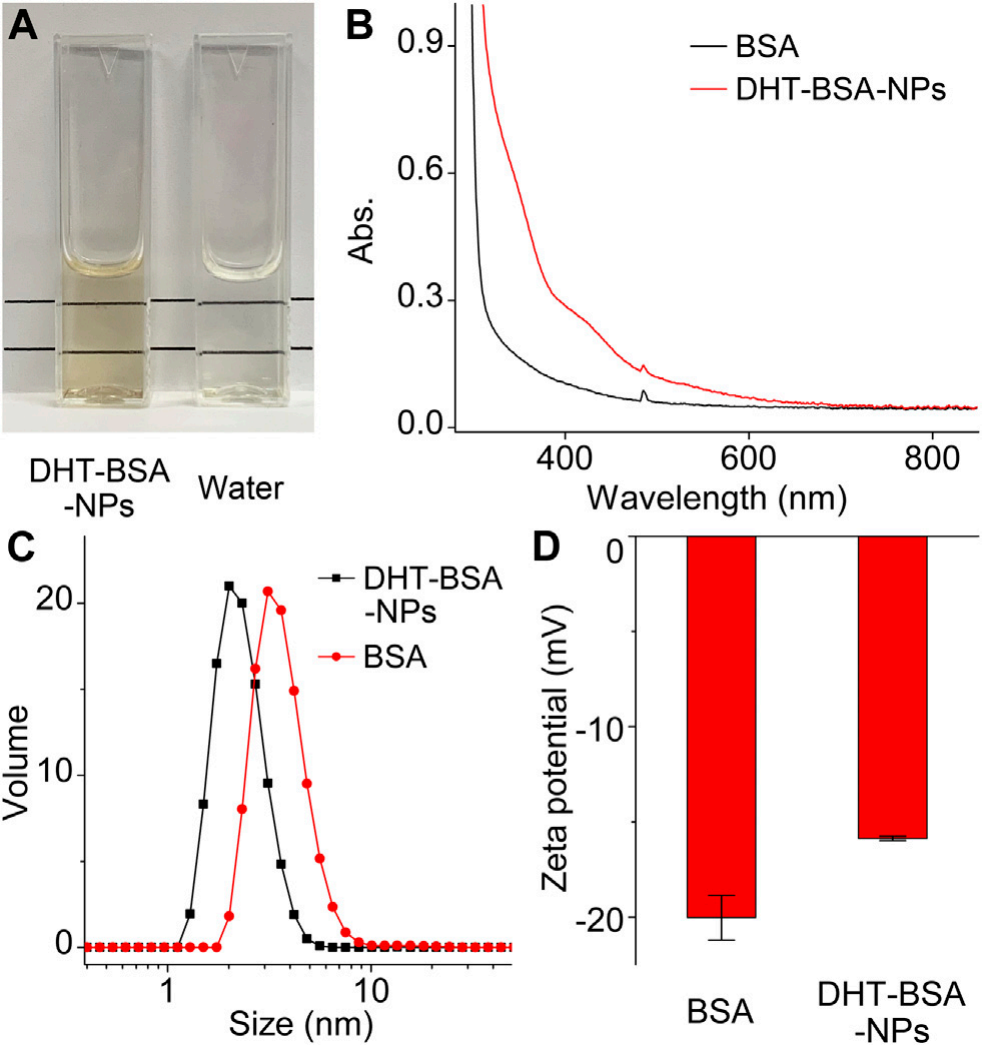
Characterization of DHT–BSA NPs. **(A)** The solubility characterization of DHT–BSA NPs. **(B)** The absorbance characterization of DHT–BSA NPs and BSA. **(C)** The hydrodynamic size characterization of DHT–BSA NPs and BSA. **(D)** The zeta potential characterization of DHT–BSA NPs and BSA.

The aqueous dispersion of the DHT–BSA NPs was firstly investigated. As displayed in Figure 1A, the formation of DHT–BSA NPs dramatically increased the solubility of DHT in aqueous solution, which is still rather clear and transparent without turbidity in comparison with pure water. The absorption spectroscopy results, as shown in Figure 1B, reveal that the overall absorption of the original BSA protein is greatly enhanced after the adsorption reaction with DHT. Meanwhile, absorption peaks appear at around 425 nm that are typical absorption bands of DHT, demonstrating that DHT–BSA NPs are successfully constructed. The impact of the DHT hydrophobic adsorption on the properties of the BSA protein was then investigated with DLS. The results given in Figure 1C reveal that the volume-weighted hydrodynamic size of nanoparticles is reasonably decreased from 3.5 to 2.2 nm after combination, which implies that DHT hydrophobic drug molecules were successfully loaded on the BSA surface and hydrophobic collapse occurred. Moreover, the light scattering profile remains nearly unchanged through the adsorption process except for the shift owing to the decreased size of the nanoparticle, suggesting that the reaction took place in a controlled manner, which did not lead to unwanted aggregates. In addition, the zeta potential of the particle surface is also changed from −20.0 mV to −15.8 mV, indicating that the surface charge density decreased after DHT adsorption (Figure 1D). The above results strongly support that the DHT molecules are effectively adsorbed onto the surface of BSA.

### Dihydrotanshinone I–Bovine Serum Albumin Nanoparticles Possess Better Biosafety Than Dihydrotanshinone I in PC12 Cells

**FIGURE 2:**
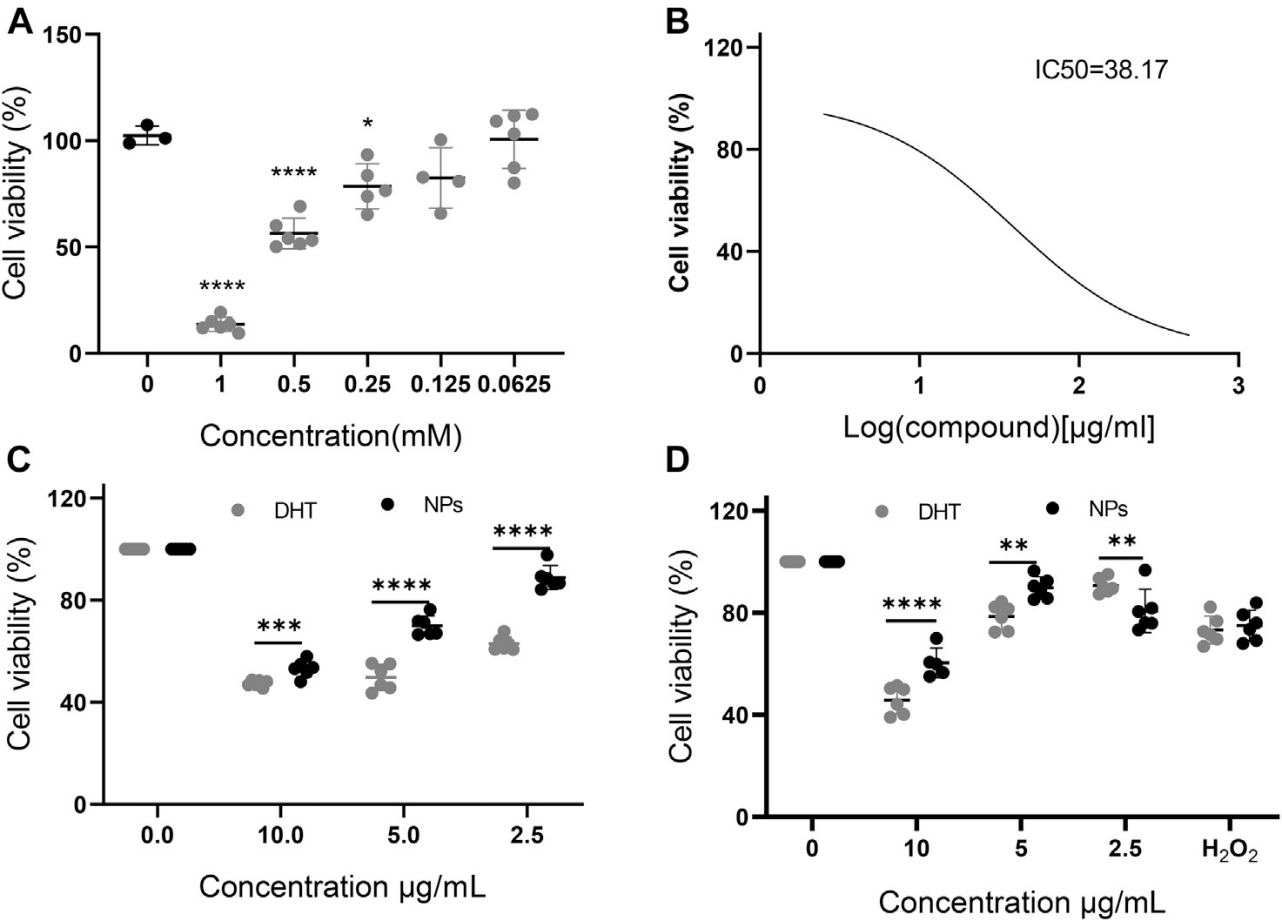
The influence of H_2_O_2_, DHT, and DHT–BSA NPs on PC12 cell viability. **(A)** Different concentrations of H_2_O_2_ act on the cell viability after 12 h. **(B)** The half-inhibitory concentration of DHT–BSA NPs on PC12 cells. **(C)** The effect of DHT and DHT–BSA NPs on cell viability. **(D)** The effect of DHT and DHT–BSA NPs on cell viability after adding H_2_O_2_. The data represent the mean ± SD (*n* = 6 in each group). *p < 0.05, **p < 0.01, ***p < 0.001, and ****p < 0.0001.

The effect of H_2_O_2_ on cell viability was measured, in which 500 µM H_2_O_2_ was used as the cell damage concentration. The cell viability measured by the CCK-8 assay showed that among the three selected concentrations of DHT and DHT–BSA NPs (2.5, 5, and 10 μg/ml), the cell viability of the DHT–BSA NPs group was significantly higher than that of the DHT group. The CCK-8 assay indicated that the percentage of viable cells following treatment with 500 µM H_2_O_2_ was 56.39% (Figure 2A). The IC50 value of DHT–BSA NPs was calculated to be 38.17 μg/ml (Figure 2B). Following the pretreatment with 2.5, 5, and 10 μg/ml DHT–BSA NPs, cell viability was 53.22, 69.95, and 88.86% (Figure 2C), respectively, which was higher than the cell viability after treatment with the corresponding concentration of DHT (Figure 2D). These results indicate that DHT–BSA NPs are able to attenuate H_2_O_2_–induced cytotoxicity in PC12 cells with satisfied biosafety.

### Dihydrotanshinone I–Bovine Serum Albumin Nanoparticles Protect PC12 Cells From H_2_O_2_ Damage by Maintaining the Superoxide Dismutase and Catalase Level

**FIGURE 3:**
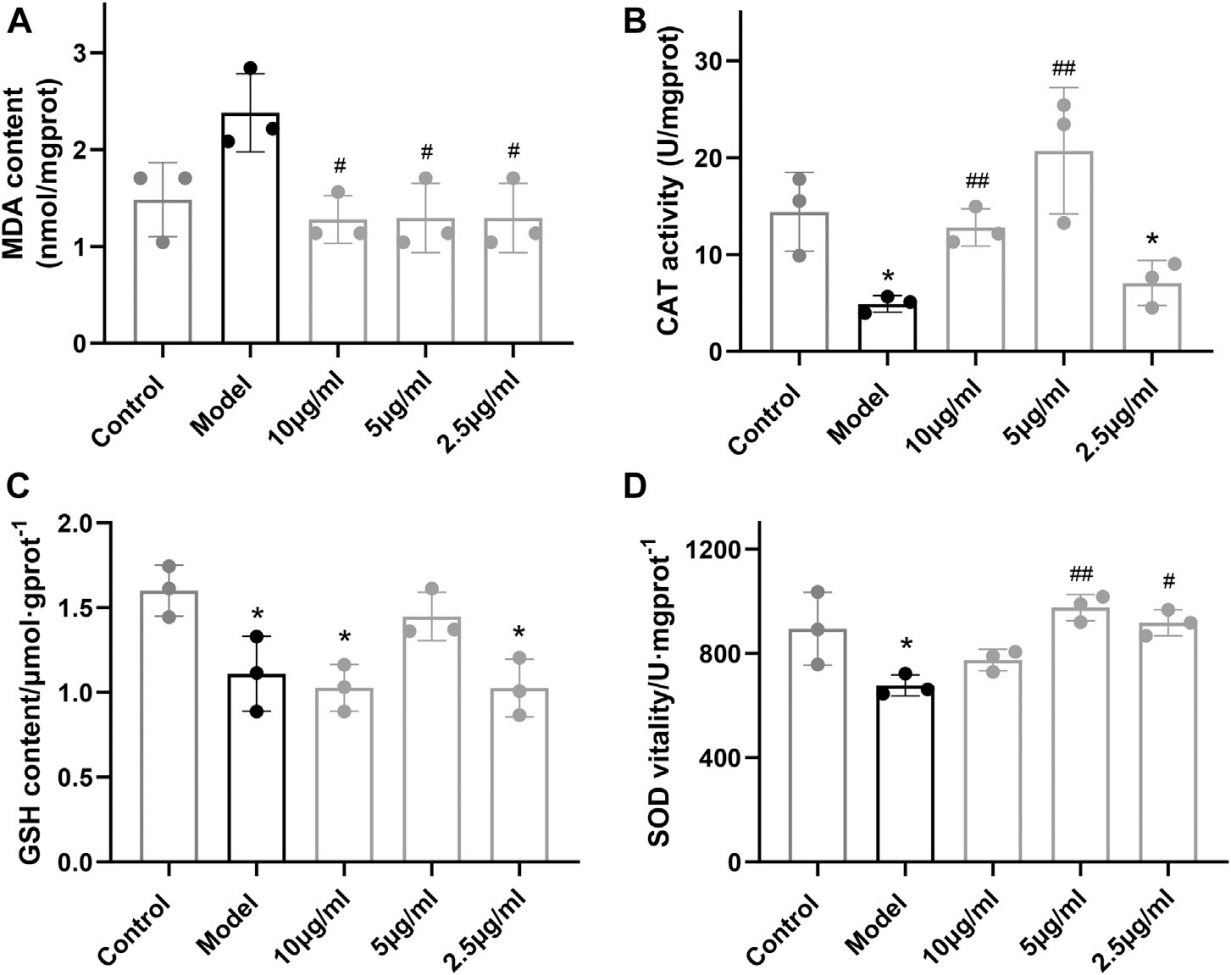
Effect of DHT–BSA NPs on SOD, CAT, GSH, and MDA levels in PC12 cells. **(A)** The effect of DHT–BSA NPs on the change of MDA content in PC12 cells after treatment with 500 μM H_2_O_2_. **(B)** The effect of DHT–BSA NPs on the change of CAT enzyme activity in PC12 cells after treatment with 500 μM H_2_O_2_. **(C)** The effect of DHT–BSA NPs on the change of GSH content in PC12 cells after treatment with 500 μM H_2_O_2_. **(D)** The effect of DHT–BSA NPs on the change of SOD enzyme activity in PC12 cells after treatment with 500 μM H_2_O_2_. The data represent the mean ± SD (*n* = 3 in each group). *p < 0.05 vs. control; ^#^ *p* < 0.05 and ^##^ *p* < 0.01 vs. model.

The activity of the antioxidant enzymes (SOD, CAT, and GSH) and the end product of oxidation (MDA) were measured in the PC12 cells. After the incubation with 500 µM H_2_O_2_, the activity levels of SOD, CAT, and GSH within cells were significantly downregulated, leading to the increased level of MDA, which implied that the cells were destroyed. In contrast, under the presence of DHT–BSA NPs, the downregulation of SOD and CAT was effectively inhibited, reducing the generation of MDA, which indicated the cell protective effect of the current DHT–BSA NPs on cells (Figure 3).

### Dihydrotanshinone I–Bovine Serum Albumin Nanoparticles Treatment Improves Neurological Dysfunction After Ischemia

**FIGURE 4:**
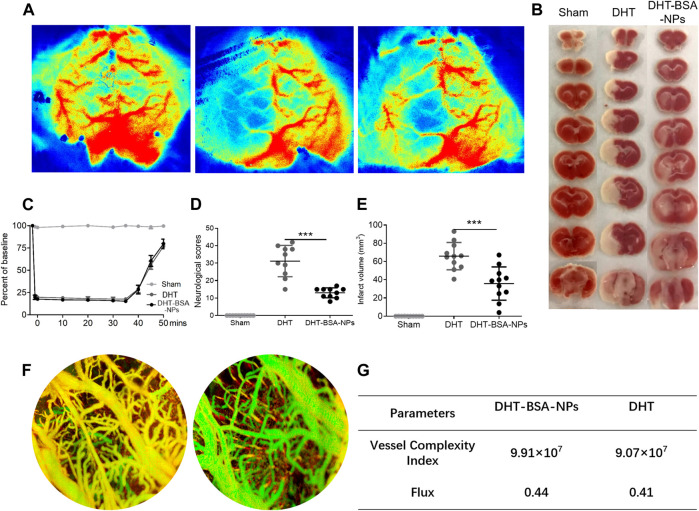
DHT–BSA NPs treatment improved the CBF of the ischemic cortex and reduced the area of cerebral infarction. **(A)** The representative pseudocolor image of CBF in the ischemic cortex after MCAO/R (left: sham; middle: DHT; right: DHT–BSA NPs). **(B)** The representative images of TTC-stained section. **(C)** Quantification of the relative CBF in the ROI (% of baseline for each animal). **(D)** MCAO/R caused significant neurological deficits. Compared with the DHT group, the DHT–BSA NPs group reduced the neurological score after MCAO/R. In contrast, rats in the sham operation group had no neurological deficits. **(E)** Quantification of the infarct volume showed that compared with the DHT group, the DHT–BSA NPs group significantly reduced the infarct volume after MCAO/R. **(F)** The OCT image of the brain tissue with DHT–BSA NPs and DHT. **(G)** OCT detects blood vessel indicators. The data represent the mean ± SD (*n* = 10 in each group). ***p < 0.05.

To evaluate the effect of DHT–BSA NPs and DHT treatments on the recovery of neurological function, the Zea-Longa score was given during the 7-day observation period. Accordingly, all the rats subjected to MCAO/R presented consistent, substantial neurological deficits 1 day after MCAO/R surgery. Although the neurological function of all the rats gradually improved during the next 6 days, the neurological deficit scores of the rats in the DHT–BSA NPs–treatment group were significantly lower than those of the rats in the DHT group after treatment, which indicated that the DHT–BSA NPs have a better effect on the therapy (Figure 4D).

### Dihydrotanshinone I–Bovine Serum Albumin Nanoparticles Treatment Improved the Cerebral Blood Flow in the Ischemic Region

It is reported that the MCAO/R can rapidly damage the cerebral microvessels and lead to hypoperfusion in the ischemic cortex and striatum; simultaneously, an emergency response system was started to compensate the depressed CBF, which included the construction of collateral circulation and angiogenesis in the impaired brain tissue (Zhang et al., 2013b). Based on this mechanism, the change of the CBF before and after the operation was determined in all of the rats. As shown in Figure 4C, in comparison with the sham group, the MCAO/R surgery induced a significant CBF decline (21.07 ± 1.95% of baseline) in the rats. After 7-day treatment, the CBF of rats was measured again. Remarkably, in comparison with DHT–treated rat, the DHT–BSA NPs–treated rats present obvious vascular signals in the ischemic hemisphere (Figures 4A,C), which suggested that DHT–BSA NPs treatment improved the recovery of the CBF in the ischemic cortex.

### Dihydrotanshinone I–Bovine Serum Albumin Nanoparticles Treatment Attenuates Infarct Volume and Neuron Loss After Ischemia

MCAO/R takes place on the right hemisphere. To further compare the efficacy of the DHT–BSA NPs, the infarct volume was measured after treatment. After 7 days, the average infarct volume of the rats treated with DHT–BSA NPs was significantly reduced (Figure 4B), in which the damage of the cortex and hippocampus was effectively repaired. In addition, OCT imaging was used to detect the changes of cerebral blood vessels after DHT–BSA NPs and DHT treatments. As shown in Figure 4F, OCT can clearly depict the blood vessels in the cerebral cortex tissue of rats. In comparison with the rat treated with free DHT, the number of microblood vessels increased significantly in DHT–BSA NPs–treated rats, which strongly support that the DHT–BSA NPs possess better therapeutic effects in terms of pathology. These results can also be verified from the vessel complexity index and flux value, as displayed in Figure 4G. In addition, the blood biochemical indexes were tested, as shown in Supplementary Table S1. After DHT–BSA NPs treatment, aspartate aminotransferase and alanine aminotransferase of the rat returned to normal significantly compared with the free DHT–treated rats (Supplementary Table S1). All these results indicated the better efficacy of the DHT–BSA NPs than free DHT.

## Discussion

Stroke is the top 10 causes of death worldwide. To date, the vast majority of pharmacological treatments fail to effectively ameliorate the sequelae of ischemic stroke. Although considerable progress had been made in understanding ischemic stroke, the development of safer, more effective drugs is of significant importance. The MCAO/R model is a typical model widely used in studies on cerebral infarction, in which the extent of ischemic damage can be controlled through sophisticated operation, as shown in Supplementary Figure S1 .

Dihydrotanshinone I (DHT) is a natural diterpenoid isolated from *Salvia miltiorrhiza* Bunge demonstrating effective anti-inflammatory properties (Supplementary Figure S2). It is accepted that inflammation plays a crucial role in the process of atherogenesis. However, its poor lipid–water partition coefficient affects its bioavailability, resulting in high-dose administration, high peripheral organ toxicity, and high clearance, which, to some extent, limits its desirable application for clinical purposes. Herein, DHT was encapsulated with BSA to enhance bioavailability. As a kind of serum albumin, BSA has been widely used in the pharmaceutical industry owing to its abundance, low cost, and ease of purification, whose biosafety including biocompatibility and biodegradability have been proved in previous studies (Elzoghby et al., 2012). As a protein with three-dimensional structure, BSA contains 583 amino acids with different side chains, which can form multiple hydrophobic binding sites. These sites show high affinity with hydrophobic molecules, especially the medium-size hydrophobic molecules (100–600 Da) (Wang et al., 2015; Khoder et al., 2016; Chen et al., 2019), such as the DHT (278.3 Da) in the current work. Therefore, the formation of DHT–BSA NPs can be mainly attributed to the hydrophobic interaction between the hydrophobic sites and DHT molecules. After adding the DHT in a tiny amount of DMSO solution into a relatively large amount of BSA aqueous solution, the solvent polarity of DHT increases instantaneously, and thus, the hydrophobic DHT molecules will quickly bind with the hydrophobic sites of BSA. This behavior will further slightly change the tertiary structure of BSA to form the hydrophobic cavity spontaneously, which ensures that more hydrophilic groups can be exposed to minimize the surface energy, as proven by the results of DLS and zeta potentials shown in Figure 1, thereby realizing a better water solubility. Based on this supramolecular self-assembly process, BSA can serve as a stable drug carrier that increases the stability of the small group of drugs.

In the cell viability experiments, it can be seen that the drug toxicity of DHT alone increases with the increase of drug concentration, while the toxicity significantly decreases after binding with BSA at the same DHT concentration. The satisfied water solubility of DHT after being encapsulated with BSA ensures the bioavailability of the drug and better exerts the anti-inflammatory effect of the drug itself, which is further proved by the SOD, CAT, and MDA levels in PC12 cells (Figure 3).

Hydrophobic drugs are difficult to deliver, which need the additives such as cosolvents of surfactants. However, the biosafety of these additives are one of the concerns, which may lead to the adverse effects in individuals. In comparison, BSA is much safer than the chemical additives, which even further improve the biosafety of DHT shown by the results of cytotoxicity experiments in Figure 2.

On the other hand, as a small molecule, DHT has a short blood half-life and will get quickly eliminated from the bodies, so it is not easy to be enriched in the lesion. In this case, using BSA encapsulated in nanosize can not only improve the water solubility of the drug but also increase the blood half-life of the drug. With these properties, the drugs are more likely to enter and accumulate within the lesions through BBB, therefore showing an enhanced bioavailability compared with free DHT in stroke treatment.

Based on the design concept of DHT–BSA NPs, the aforementioned problems can be addressed satisfactorily. According to the results of *in vivo* treatment experiments (Figure 4), a significant recovery of blood flow was observed after 7 days through laser-Doppler spectrophotometry after MCAO/R, which is mainly attributed to the protective effect of DHT–BSA NPs on neuronal cells from oxidative stress and inflammation during ischemia. From the OCT imaging, the microblood vessels of DHT–BSA NPs treatment rats are well protected, which also implied the recovery of blood flow. Compared with the free DHT group, the DHT–BSA NPs further significantly reduced the area of cerebral infarction, demonstrating their satisfied therapeutic effect for ischemic stroke. Under such a therapeutic mechanism, the neurological behavioral score of MCAO/R rats changed significantly and the blood biochemical indexes were also recovered. These results displayed the satisfied drug efficacy of the current DHT–BSA NPs.

It should be noted that DHT has also been reported to be used in tumor treatment as well as in the prevention and treatment of atherosclerosis, which bodes well for the theoretical basis of this study for the therapeutic application of DHT in brain-related diseases and highlights the therapeutic potential of DHT for other cardiovascular and cerebrovascular diseases. The current study proposed a feasible strategy for the clinical use of poorly water-soluble drug DHT, which introduces BSA to form DHT–BSA NPs, remarkably enhancing the biocompatibility, bioavailability, and biosafety of DHT. Therefore, it is believed that this approach will pave a new way for expanding the application and evaluation of DHT in clinics.

## Conclusion

In summary, the solubility and biocompatibility of poorly water-soluble drug DHT was successfully enhanced through the introduction of BSA. Through the oxidative damage experiment of H_2_O_2_ on PC12 cells, the resultant DHT–BSA NPs can significantly increase the viability of H_2_O_2_–oxidatively damaged cells in comparison with the free DHT. By measuring SOD and CAT enzyme activities, and GSH and MDA contents in cells, it is further proved that DHT–BSA NPs can enhance the antioxidant activity of DHT. In the rat MCAO/R model, DHT–BSA NPs can be readily delivered in to the stroke area due to the drug delivery behavior of the nanosystem, which successfully reduces the area of cerebral infarction and lowers the neurological score through protecting the neuron cells and promoting the recovery of blood flow. Therefore, through the current strategy, DHT–BSA NPs showed an enhanced bioavailability compared with free DHT and a successful penetration into the central nervous system for stroke therapy, demonstrating their application potential in cardio–cerebrovascular diseases.

## Data Availability

The original contributions presented in the study are included in the article/Supplementary Material; further inquiries can be directed to the corresponding authors.

## Abbreviations

BBB, Blood brain barrier; BSA, Bovine serum albumin; CAT, Catalase; CBF, Cerebral blood flow; DHT, Dihydrotanshinone I; DHT–BSA NPs, DHT–BSA nanoparticles; GSH, Glutathione; MCAO/R, Middle cerebral artery occlusion/reperfusion; MDA, Malondialdehyde; OCT, Optical correlation tomography; SOD, Superoxide dismutase.
