# Supplementary material for: Enhanced Bioavailability of Dihydrotanshinone I–Bovine Serum Albumin Nanoparticles for Stroke Therapy
Source: Front Pharmacol. 2021 Aug 31;12:721988. doi: 10.3389/fphar.2021.721988 (PMC8438562; doi:10.3389/fphar.2021.721988)
Supplement: Supplementary file 1 [file DataSheet1.docx]

Supplementary Material

Enhanced Bioavailability of Dihydrotanshinone I-BSA Nanoparticles for Stroke Therapy

**Yanru Ren^1#^**, **Yicheng Feng^1#^**, Kunyao Xu^1^, Saisai Yue^1^, Tiantian Yang^1^, Kaili Nie^1^, Man Xu^1^, Haijun Xu^1^, Xin Xiong^2^, Fabian Körte^2^, Mike Barbeck^3^, **Peisen Zhang^1^*** and **Luo Liu^1^***

1. Beijing Advanced Innovation Center for Soft Matter Science and Engineering, College of Life Science and Technology, Beijing University of Chemical Technology, Beijing 100029, China.
2. NMI Natural and Medical Sciences Institute at the University of Tübingen, Markwiesenstr. 55, 72770 Reutlingen, Germany.
3. Institute of Material Science and Technology, Technical University of Berlin, Hardenbergstrasse 40, Sekr. BA3, 10623 Berlin.

# Both are the first authors

* Email of the corresponding authors should be addressed: liuluo@mail.buct.edu.cn and zhangps@iccas.ac.cn

## 1. Supplementary Figures

##
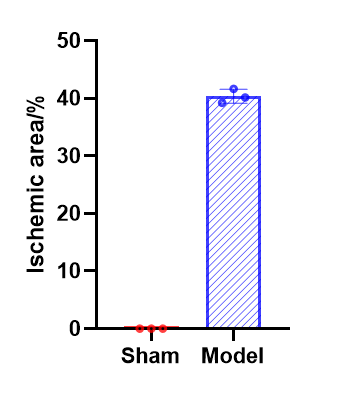


## Supplementary Figure 1. The statistical results about the cerebral ischemic area of the rMCAO rat models and sham-operated rats (n = 3).

##



## Supplementary Figure 2. The molecular formula of Dihydrotanshinone I.

## 2. Supplementary Tables

## Supplementary Table 1. Changes of serum AST and ALT after DHT-BSA-NPs or DHT treatment.


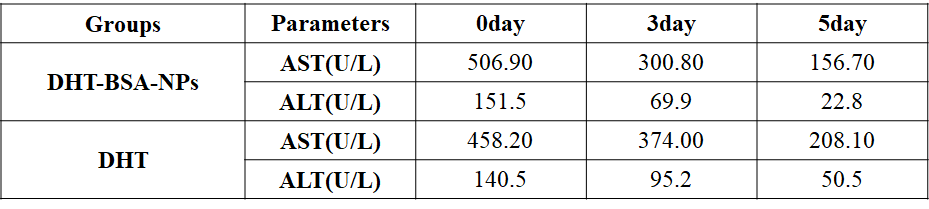


Note: AST reference value is 0-37U/L, ALT reference value is 0-40U/L.
